# Spontaneous Cell Detachment from Temperature Gradients: Getting the Method Ready for Antimicrobial Drug Testing at Cell Culture Level

**DOI:** 10.3390/s25092902

**Published:** 2025-05-04

**Authors:** Csongor Tibor Urbán, Soroush Bakhshi Sichani, Gabriela Ueda Modaffore, Christ Glorieux, Jonas Gruber, Derick Yongabi, Minne Paul Lettinga, Patrick Wagner

**Affiliations:** 1 Laboratory for Soft Matter and Biophysics ZMB, Department of Physics and Astronomy, KU Leuven, Celestijnenlaan 200 D, B-3001 Leuven, Belgium; csongortibor.urban@kuleuven.be (C.T.U.); pavlik.lettinga@kuleuven.be (M.P.L.); 2 Department of Fundamental Chemistry, Institute of Chemistry, University of São Paulo, Prof. Lineu Prestes Ave., 748, São Paulo 05508-000, SP, Brazil; jogruber@iq.usp.br; 3 Biomolecular Systems and Processes IBI-4, Institute of Biological Information Processing, Research Center Jülich, Wilhelm-Johnen-Strasse, D-52428 Jülich, Germany

**Keywords:** cell-based biosensors, *S. cerevisiae*, heat-transfer method HTM, temperature gradients, spontaneous cell detachment, microfluidic aspect ratio, Amphotericin B, povidone-iodine

## Abstract

Spontaneous cell detachment describes an effect in which eukaryotic cells first sediment onto a heated chip and then detach from it spontaneously and collectively after a sharply defined dwell time *t_d_*. This behavior is triggered by the temperature gradient between the chip and the colder supernatant liquid. Notably, *t_d_* allows distinguishing between different yeast strains and cancer-cell lines. At the same time, it also varies in the presence of nutrients and cytotoxins, suggesting an added value of this method for pharmacological studies. In the present work, we study the role of fluid convection on the detachment of yeast cells experimentally and by simulations using a sample compartment with a variable aspect ratio. Hereby, we found that the absolute chip temperature, the strength of the temperature gradient and the number of cells inside the sample compartment all affect the dwell time *t_d_*. To demonstrate the concept, we show that the spontaneous-detachment method can measure the impact of an antibiotic and an antiseptic drug on yeast cultures and corroborate this with reference assays.

## 1. Introduction

Bio and chemosensors detect a wide variety of targets, from small molecules to whole cells. They typically combine a receptor element with a transducer to obtain quantitative signals to indicate the concentration of the target elements within a sample. Many of these transducers rely on optical, electronic, electrochemical and microgravimetric sensing principles. Comprehensive review articles can be found in Refs. [1,2,3,4]. The present work will address a thermal transducer known as the heat-transfer method (HTM). This method measures the interfacial thermal resistance (ITR) between a solid chip, often functionalized with target-selective receptors, and the liquid sample. Although ITR is easy to measure, ab initio calculations are complex, and most numerical results are derived from molecular dynamics simulations [5]. HTM was introduced in 2012 in the context of DNA mutation analysis [6]. Since then, numerous applications have been developed, including small-molecule detection of PFAS compounds in water and soil, which is a recent example [7]. HTM enables the detection of bacteria and human cells in complex samples [8,9,10,11] and can also identify Noro and SARS-CoV-2 virus particles in bodily fluid samples by integrating the HTM readout with suitable receptors [12,13]. An overview of all applications utilizing HTM can be found in Ref. [14]. The technique can also measure ITR with chips without a receptor coating, allowing it to probe ITR changes when a cell layer is present at the interface between the chip and the supernatant.

The spontaneous cell-detachment effect, first reported by Yongabi et al. [15], describes a collective phenomenon observed in eukaryotic cells, including yeast and cancer cells. The cells initially settle onto a heated chip, and after a specific dwell time, they detach collectively with a characteristic detachment time *t_d_*. This effect is thermally driven and differs from cell detachment in adherent cancer-cell lines and enzymatic cell detachment [16,17]. Depending on the chip temperature and the cell type, typical *t_d_* values range between 10 min and 2 h. Importantly, detachment occurs without external triggers and *t_d_* is sharply defined. The effect is highly reproducible with minimal variability [15,18]. Although the effect can be probed with multiple transducers, measuring the thermal resistance *R_th_* between the chip and the supernatant liquid provides high signal-to-noise ratios. Impedance spectroscopy and microgravimetry using a quartz–crystal microbalance are also viable options, though they offer a lower signal-to-noise ratio [18]. According to live-cell imaging with confocal fluorescence microscopy on HeLa cells, the separation between the chip and the cells is below 100 nm after detachment [15]. However, this distance is large enough to allow cells to move freely via Brownian motion across the chip surface, significantly reducing the cell–chip interaction.

Multiple factors may contribute to the detachment of cells from the chip surface. Thermophoretic forces naturally drive particles and cells from warmer to cooler regions [19,20,21]. In addition, cell membrane protrusions, known as blebs, can form on the heated side of the cells, a process involving a reorganization of the cytoskeleton [22]. Bernoulli forces resulting from convective fluid movement are a third option, but previous modeling data suggest that the resulting forces are possibly too weak to lift sedimented cells [15]. A fourth possibility is an increase of the Archimedes forces due to the thermal expansion of the cells upon contact with the chip. However, the expansion coefficients reported in the literature suggest this effect is minimal [23]. Cell proliferation can be ruled out, as detachment occurs long before proliferation can take place. Additionally, no signs of proliferation were observed in live-cell imaging [15]. In summary, the most plausible explanation to date involves a combination of membrane blebs, thermophoretic forces and convection. However, the reason why this effect occurs only after a certain detachment time *t_d_* remains a significant unresolved question.

Besides the fundamental issue of the driving mechanisms, the current knowledge on the effect consists mainly of empirical observations [15,18]. Together, they indicate that the method can be used for cell identification, checking their metabolic status and probing their response to cytotoxic compounds. Hence, one may think of applications in drug-efficacy studies and antimicrobial-resistance testing. A review of existing techniques can be found in Ref. [24]. The following is known in summary: (i) The dwell time *t_d_* decreases with increasing chip temperature *T*_1_ according to an exponential scaling law. (ii) For a given value of *T*_1_, *t_d_* depends on the cell type, as observed for cancer-cell lines and different yeast strains. Yeast cells with functional flocculation genes detach faster than yeast with altered *FLO* genes. (iii) Nutrients accelerate detachment in a concentration-dependent way, suggesting a role of metabolic processes in the effect. (iv) Blebbistatin and DMSO (dimethyl sulfoxide) cause a concentration-dependent lengthening of *t_d_*. Blebbistatin blocks the activity of the motor protein myosin and prevents bleb formation, while DMSO increases cell membrane porosity and exerts oxidative stress at higher doses [25,26]. (v) At low chip temperatures (e.g., 25 °C) and in the presence of nutrients, yeast cells show sustained oscillations of the *R_th_* signal with a defined periodicity that resembles glycolytic oscillations [27].

When comparing the data in Refs. [15,18], it is evident that, for the same cell type (*S. cerevisiae*) and chip temperature, dwell times *t_d_* can vary. The difference in *t_d_* occurs when probing the effect in sample compartments with differing dimensions as quantified by the aspect ratio *Γ* (height-to-diameter ratio), a key parameter in microfluidics [28]. Adjusting the height affects the temperature gradient and internal temperature distribution for a given chip temperature. Since *Γ* determines whether heat transport is governed by conduction or convection, it is crucial to reassess whether convection is the physical driver of cell detachment. Additionally, it may facilitate the exchange of intercellular signaling molecules [29,30]. This work aims to define the most sensitive geometry to detect this effect. To achieve this, we will use a sample compartment with an adjustable inner height covering a broad range of *Γ*. Experimental and computational results will demonstrate that convection is present in all cases where cell detachment occurs. The detachment time *t_d_* for yeast cells will be studied as a function of temperature, temperature gradient, cell concentration and the aspect ratio *Γ*. Finally, we will show that with the optimized device settings, it is straightforward to employ thermal detachment as a facile technique to study the efficacy of antimicrobial compounds. One antimicrobial used is Amphotericin B, an established drug for treating fungal infections (e.g., *Candida albicans* and *S. cerevisiae*). This compound binds to ergosterol in the yeast cell membrane [31,32,33,34]. The second drug is povidone-iodine, an antiseptic of which iodine ions can easily permeate cell walls and membranes, leading to the oxidation of proteins, nucleic acids and fatty acids [35,36,37].

## 2. Materials and Methods

### 2.1. HTM Sensing Device with a Variable Aspect Ratio

**Figure 1:**
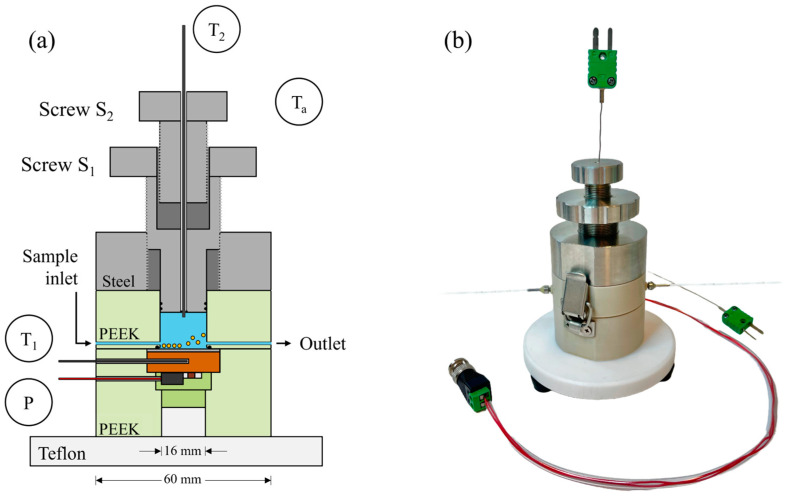
(**a**) Schematic cross-section of the heat-transfer device with all components drawn to scale. The device can be placed inside a temperature-controlled incubator that stabilizes the background temperature *T_a_* to a predefined value. The chip was heated from its underside to a temperature *T*_1_ using the power resistor *P*, and the temperature *T*_2_ inside the compartment was measured with a thermocouple. The inner height *h_i_* can be regulated between 2.0 mm and 16.0 mm (aspect ratios 0.125 ≤ *Γ* ≤ 1.00) using screw *S*_1_. The position of the thermocouple tip for measuring *T*_2_ was adjusted with the screw *S*_2_. (**b**) Photographic image of the HTM device. The sedimentation and detachment of cells are identified via changes in *T*_2_, respectively, in the thermal resistance signal *R_th_*.

The device is depicted in Figure 1a as a schematic cross-section, and Figure 1b shows a photographic image. *T*_1_ is the chip temperature, *T*_2_ is the temperature inside the sample compartment and the thermal resistance is calculated as *R_th_* = (*T*_1_ − *T*_2_) *P*^−1^, where *P* denotes the applied heating power [6,14]. The sample compartment, made of PEEK (polyether ether ketone), has a cylindrical shape with a 16 mm diameter. It is on top of a round sensor chip made of polished stainless steel without further modifications (alloy AISI 304, 22 mm diameter, 1.0 mm thickness, Testas NV, Wommelgem, Belgium). The sensor chip was heated at its underside to a predefined temperature *T*_1_ using a power resistor (Farnell MPR-20, 47 Ω, Farnell, Machelen, Belgium). Combined with a copper disk (23.0 mm diameter, 7.0 mm height), this enabled a homogeneous temperature distribution. The temperature *T*_1_ inside the copper disk was measured with an embedded thermocouple (type K, 0.5 mm diameter, TC Direct, Nederweert, The Netherlands). The chip was pressed mechanically onto the copper disk with a thin interlayer of heat-conducting silver paint to minimize the roughness effects. The inner height *h_i_* of the sample compartment can be regulated between 2.0 and 16.0 mm, corresponding to aspect ratios between 0.125 and 1.0. This was enabled by a piston-like top lid, sealed with O-rings, which can be moved up and down with screw *S*_1_ (AISI 304, pitch of 1.00 mm per rotation). Screw *S*_2_, with the same alloy and pitch, moves relative to screw *S*_1_ and features a channel through which a second thermocouple runs down to the sample compartment. This way, the tip with the thermocouple junction can be positioned at any point along the central axis of the compartment. The thermocouple channel was sealed from below with an additional O-ring.

The sample inlet and outlet have openings of 1.5 mm^2^, and their central part was placed 2.5 mm above the chip surface in all measurements to keep the sedimentation time short. The inlet and outlet are integrated into rotatable PEEK cylinders (embedded in the upper PEEK body) to freely regulate their height above the chip surface between 0.5 mm and 6 mm. The lower PEEK body, which houses the copper disk and power resistor, minimizes heat loss in the lateral directions, while downward heat loss was further reduced by a press-fit Teflon disk (20.0 mm thickness in its central part and 10 cm diameter). All components are chemically resilient and can be sterilized.

HTM measurements with this device were performed using a custom-built module designed to regulate and monitor temperatures via a closed-loop feedback system [38]. This device functions as a dual-channel voltage source, providing up to 50 W per channel, and serves both as the primary power supply and temperature control unit. A LabVIEW-based PID controller was employed to maintain the *T*_1_ chip temperatures within the range of 25 to 42 °C. The chosen PID parameters were 1–6–0 in agreement with Ref. [38].

### 2.2. Heat and Mass Transfer Simulations with COMSOL

To understand the role of temperature distribution and convective flow profiles inside the sample compartment in the detachment effect, the system was studied with finite element modeling with COMSOL Multiphysics software Version 6.2 (COMSOL Inc., Burlington, VT, USA), see Ref. [39] for the weblink. To simplify the 3-dimensional device into a 2-dimensional geometry, a 2D-axisymmetric COMSOL model was built, as shown in Figure S1 of the Supporting Information S1. This made the simulations computationally leaner without compromising accuracy. The simulated materials, including their thermophysical properties, are listed in Table 1.

The temperature of the copper block was set to a defined temperature of *T*_1_, and it was covered with a stainless-steel chip. The flow cell height was parametrically adjusted by varying the aspect ratio with the inner height from 2.0 to 8.0 mm while keeping the base radius at 8.0 mm. Fourier’s law, see Equation (1), was used to model the heat transport in domains 1–6, see Supporting Information S1, as follows:

(1) $$\kappa \nabla^{2}T=0,$$

where *κ* represents the thermal conductivity (W m^−1^ K^−1^) and *T* represents temperature (K). A density gradient will form in the heated water domain due to a temperature gradient and a gravitational body force. This density gradient, calculated by the following Equation (2), will contribute to heat transport across the geometry, which can be calculated by the Rayleigh number, as described in Ref. [41]:

(2) $$\frac{g\beta \left(T_{1}-T_{2}\right)L^{3}}{\alpha v}=0,$$

where *g* represents the gravitational constant (N kg^−1^), *β* the thermal expansion coefficient (K^−1^), *T*_1_ − *T*_2_ the temperature difference between the bottom and top plate (K), *L* the distance between these two plates (m), *α* the thermal diffusivity (m^2^ s^−1^), and *v* representing the fluid’s kinematic viscosity (Pa s). Laminar flow modeling is based on the Navier-Stokes equations that consider the energy balance, the momentum balance and the mass balance of the sample domain, see Equations (3)–(5), as follows:

(3) $$\rho C_{p}u\cdot \nabla T-k\nabla^{2}T=0,$$

(4) $$\left(u\cdot \nabla \right)u=\nabla \cdot \left[-pl+v\left(\nabla u+{\left(\nabla u\right)}^{T}\right)-\frac{2}{3}v\left(\nabla \cdot u\right)I\right]+g,$$

(5) $$\nabla \cdot \left(\rho u\right)=0,$$

where *ρ* represents the density (kg m^−3^), *C_p_* the isobaric heat capacitance (J kg^−1^ K^−1^), *u* the velocity field (m s^−1^) and *I* the unity tensor. These equations were coupled via the non-isothermal laminar flow interface, selected for modeling because the temperature difference between the bottom and top plate (at ambient temperature *T_a_* = 20 °C) stays within 13 °C.

### 2.3. Spontaneous Cell-Detachment Measurements with S. cerevisiae

For the initial characterization of the device, pure Milli-Q water was used to exclude any salt deposits on the stainless-steel chip. For measurements with yeast cells, 1 × PBS buffer was used containing 137 mM NaCl, 2.7 mM KCl, and 10 mM of phosphates, resulting in pH 7.4 at room temperature. One PBS tablet (Sigma Aldrich, St. Louis, MO, USA) was dissolved per 200 mL of Milli-Q water. *S. cerevisiae* was obtained as dried yeast from Dr. Oetker (Bielefeld, Germany). Commercially available yeast was chosen for its low cost, ease of cultivation and consistent quality, making it suitable for method development. The yeast pellets were resuspended in 1 × PBS buffer by vortexing for 10 s. OD600 measurements (Ultrospec 2100 pro, Biochrom Ltd., St. Albans, UK) confirmed that the expected concentrations, calculated from the dissolved cell mass, were consistent with the observed data. In this article, all concentrations are expressed in mg mL^−1^ ranging from 1 mg mL^−1^ up to 25 mg mL^−1^ (4.28 × 10^6^ cells to 1.07 × 10^8^ cells per mL). Measurements were verified by manual particle counting using a Neubauer-improved counting chamber (Paul Marienfeld GmbH and Co. KG, Lauda-Königshofen, Germany). To inject the dissolved yeast samples into the HTM sensor setup, an automated syringe pump (ProSense NE 500, Oosterhout, The Netherlands) was set to a flow rate of 5.0 mL min^−1^ for 2.0 min. The injected volume of 10 mL exceeds the inner volume of the sample compartment by a factor of 3.6 (2.81 mL when the top lid is at the highest used position, *h_i_* = 14 mm). During sedimentation, dwelling and detachment, no flow was imposed inside the sample compartment. Between each measurement, the sensor device was thoroughly cleaned with 70% ethanol and dried with nitrogen gas. To improve the accuracy, all measurements were performed with the sensor device and the flow pump enclosed in a temperature-controlled incubator (model R-TH-50, Labtech Instrument Co., Ltd., Dongguan, China). The ambient temperature *T_a_* inside the incubator was set to 20.0 °C unless mentioned otherwise.

### 2.4. Drug Exposure Measurements with Amphotericin B and Povidone-Iodine

For detachment measurements in the presence of an antimicrobial, first *Amphotericin B* (AmpB) was used (Product code A2942, Sigma-Aldrich, St. Louis, MO, USA) dissolved in Milli-Q water. The samples were prepared with a volume of 10 mL at the concentrations of 15 mg yeast mL^−1^ and 10 µM AmpB, as well as without AmpB as a negative control. The minimum inhibitory concentration (MIC) of AmpB for *S. cerevisiae* is in the order of 0.03 to 1.0 mg L^−1^, and 10 µM corresponds to 9.24 mg L^−1^ [42]. The yeast suspensions (in 5.0 mL 1 × PBS) and the AmpB solutions (in 5.0 mL Milli-Q) were prepared separately. After mixing, they were incubated at 30 °C for 48 h to let AmpB interact with the cells. The detachment measurements were started by injecting the samples into the HTM device. These measurements were repeated with polystyrene (PS) microbeads (5 µm diameter, density 1.05 g cm^−3^, Merck, Darmstadt, Germany), which are similar in size to yeast but are assumed to be insensitive to AmpB. Finally, quartz–crystal microbalance measurements were carried out on yeast cells (QCM-D E4, Biolin Scientific, Gothenburg, Sweden) to assess whether AmpB affects their viscoelastic properties.

For testing the impact of the antiseptic drug povidone-iodine (PovI), a complex of polyvinylpyrrolidone with I_3_^−^ ions (iso-Betadine^®^ Dermicum 10%, Viatris Health-care, Hoeilaert, Belgium) was used, which contains 10 g of the PovI complex per 100 mL. Yeast samples of 10 mg mL^−1^ (total volume of 10 mL) were prepared in 1 × PBS buffer (negative control) as well as in 1 × PBS supplemented with 1, 10 and 50% *v*/*v* iso-Betadine. Given the fact that the active compound in this commercial product was already tenfold diluted, these samples are addressed as 0.1, 1.0 and 5.0%. Before the HTM measurements, the samples were incubated for 30 min at room temperature. Longer incubation times (such as with AmpB) were unnecessary due to the fast biocidal action of PovI. These measurements were also repeated with PS microbeads instead of yeast cells and consolidated with QCM-D data.

### 2.5. Reference Methods

To visualize the yeast cells with and without exposure to AmpB and PovI, images were obtained at room temperature using a widefield microscope (DM 750M, Leica Microsystems, Heerbrugg, Switzerland) coupled with a CCD camera (Leica MC170 HD). For cell imaging, a 100×/0.85 NA objective was used (N Plan Epi 100×/0.85 from the same manufacturer). The corresponding data is provided in Supporting Information S3. Based on our current understanding, *t_d_* of spontaneous cell detachment is directly linked to metabolic activity. Therefore, a resazurin reagent (purity > 99%, Acros Organics, Thermo Fisher Scientific, Geel, Belgium) was used to assess cell viability. Respiratory reactions in the mitochondria cause the reduction of resazurin (blue color) to resorufin (pink color). The color change serves as an indicator of viable, metabolically active cells. Resazurin solutions (15 µM) were prepared in 1 × PBS buffer (pH 7.4). Cell suspensions were incubated at 30 °C for 30 min. Treated cells were incubated in 10 µM AmpB or 5% PovI, respectively. After incubation, cells were centrifuged at 2000× *g* for 5 min at 4 °C. Centrifugation was performed three times, and cells were resuspended in 1 × PBS buffer. Finally, the cell suspensions were incubated with the resazurin solution (10% *v*/*v*) for 6 h. Cell viability assay can be used to confirm that cells are metabolically active after the detachment process (see Supporting Information S4). Since buoyancy and surface interactions could influence detachment, the density and contact angle of all fluids and substrates were measured using a density meter (Densito, Mettler Toledo N.V., Zaventem, Belgium) and a contact angle device (DataPhysics, OCA 25, Filderstadt, Germany). The corresponding data can be found in Supporting Information S5.

## 3. Results

### 3.1. Thermal Resistance of the Device for Different Aspect Ratios

**Figure 2:**
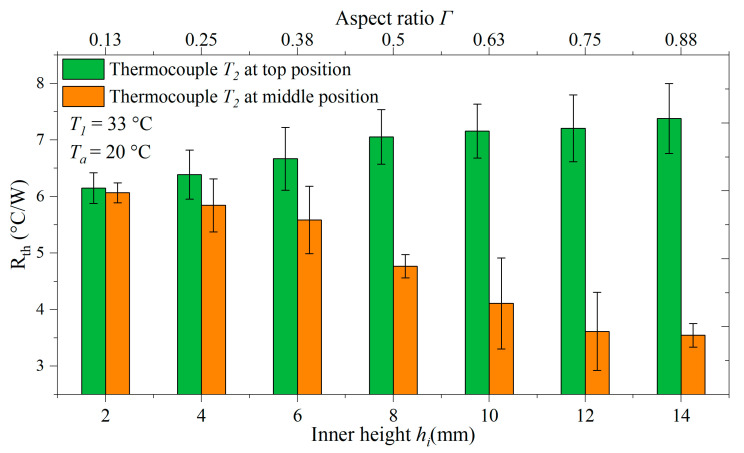
Thermal resistance *R_th_* of the device for different inner heights *h*_i_ (lower *x*-axis) and the corresponding aspect ratio *Γ* indicated at the upper *x*-axis. In all cases, the sample compartment was filled with Milli-Q water, and the ambient temperature was *T_a_* = 20.0 °C. Bars in green represent the data with the tip of the *T*_2_ thermocouple positioned at 1.0 mm distance underneath the top lid. The data in orange are for measurements in which the thermocouple tip was exactly in the middle of the compartment, at *h_i_*/2 underneath the lid. For inner heights above 10 mm, the *R_th_* values with the thermocouple at the middle position are half of those measured when using the top position. All data points are the average of three independent measurements, and the width of the error bars is the standard deviation.

Prior to biological measurements with yeast cells, the device was characterized by measuring its thermal resistance signal according to *R_th_* = (*T*_1_ − *T*_2_)/*P* using Milli-Q water. For these experiments, *T*_1_ was set to 33 °C and *T_a_* to 20 °C. *R_th_* entails the ITR between the chip and the liquid and the thermal resistance of the liquid column. Note that the heating power *P* does not completely pass through the chip–liquid interface, but a major fraction is dissipated into the environment. Details are discussed in Section 3.1, where the thermal conductivity of water was determined in comparison to the literature data. Figure 2 shows the corresponding data for inner heights from 2.0 to 14.0 mm with the tip of the *T*_2_ thermocouple at the top position (1 mm underneath the top lid) and the middle position at *h_i_*/2.

**Figure 3:**
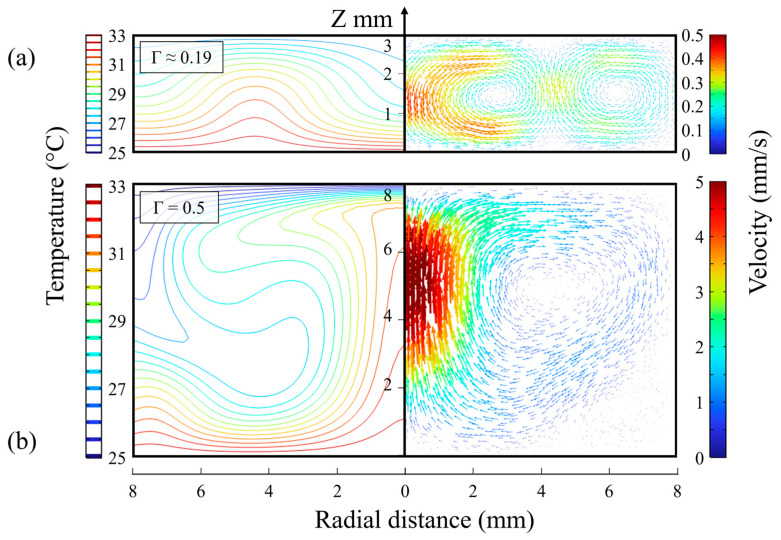
(**a**) COMSOL simulations of the temperature distribution (**left**) and the velocity profile (**right**) inside the sample compartment for the inner height *h_i_* = 3.0 mm (*Γ* = 0.19) at the chip temperature *T*_1_ = 33.0 °C and ambient temperature *T_a_* = 20 °C. Panel (**b**) shows the corresponding data for *h_i_* = 8.0 mm, *Γ* = 0.5. Note that the temperature distribution along the central axis, where the *T*_2_ thermocouple was located, deviates from strict linearity. The convective flow pattern has a maximum velocity *v_max_* of 0.43 mm s^−1^ for *h_i_* = 3.0 mm and up to 5.48 mm s^−1^ in the case of *h_i_* = 8.0 mm.

The nearly linear increase of *R_th_* with increasing *h_i_* for the top position relates to the increasing thermal resistance of the taller water column. For *h_i_* = 2.0 mm, the data points have identical values as expected, while for larger inner heights, such as *h_i_* = 14.0 mm, the middle position was expected to yield half of the *R_th_* value for the top position. In good approximation, *R_th_* values of 3.55 +/− 0.21 °C/W (middle) versus 7.38 +/− 0.62 °C/W were obtained when choosing *h_i_* = 14.0 mm. As seen in Figure 2, there was a deviation from the linearity of *R_th_* when looking at intermediate *h_i_* values. This deviation can be understood when the convective motion of the fluid is considered. Therefore, the internal temperature and velocity profiles of the sample compartment were studied with the COMSOL heat and mass transfer module. The data for the given condition (*T*_1_ = 33 °C, *T_a_* = 20 °C) and *h_i_* = 3.0 mm, respectively, 8.0 mm, are shown in Figure 3. Other settings of *T*_1_ and *h_i_* utilized within this article were modeled as well, and the corresponding data are provided in Supporting Information S1. In addition, the Rayleigh numbers were calculated for each condition (the background is described in Ref. [41]). From this, it was concluded that convection was always in the laminar regime.

To assess the influence of convection on the obtained *R_th_* values, the thermal conductivity of water was measured as the reference material. The settings were *T*_1_ = 33 °C and *h_i_* = 10.0 mm. We used an algorithm developed by Stilman et al. to determine the heating power *P_d_* that dissipates to the ambient without passing the interface between the chip and the sample compartment [40]. *P_d_* was hereby derived from measuring *P* and *T*_2_ with an empty air-filled sample compartment, and the thermal conductivity of the air column (*κ*_air_ = 0.0262 W m^−1^ K^−1^, see Table 1) was taken into account. Then, the measurement was repeated after filling the compartment with Milli-Q water. Taking the dimensions of the water column into account, a nominal *κ* value of 1.091 W m^−1^ K^−1^ was obtained. This value exceeds the literature value of Table 1 by a factor of two, indicating that convection strongly enhances heat transfer. For a systematic study on this effect, including different aspect ratios and vessel shapes, we refer to recent work by Hartmann and co-authors [43].

The contribution of convection to heat transfer can be effectively suppressed by inverting the device, ensuring that the heated chip surface faces downward. The corresponding data for *h_i_* = 10.0 mm and chip temperatures *T*_1_ from 25 °C up to 55 °C are provided in Supporting Information S2. Following the algorithm of Ref. [40], *T*_2_ and *P* were measured with an empty sample compartment to determine again the dissipated power *P_d_*. For *T*_1_ = 35 °C, *κ*_H20_ = 0.652 W m^−1^ K^−1^, which is in close agreement with its literature value, see Table 1. For 1 × PBS, the same analysis yielded *κ*_1×PBS_ = 0.523 W m^−1^ K^−1^. Thermal conductivity values of 1 × PBS are only available in its frozen state [44], but a decreased thermal conductivity, compared to H_2_O, is plausible given the formation of hydration shells around the ions that decrease the mobility of H_2_O molecules in diffusion [45].

### 3.2. Temperature-Dependence of the Detachment Time of Yeast Cells

**Figure 4:**
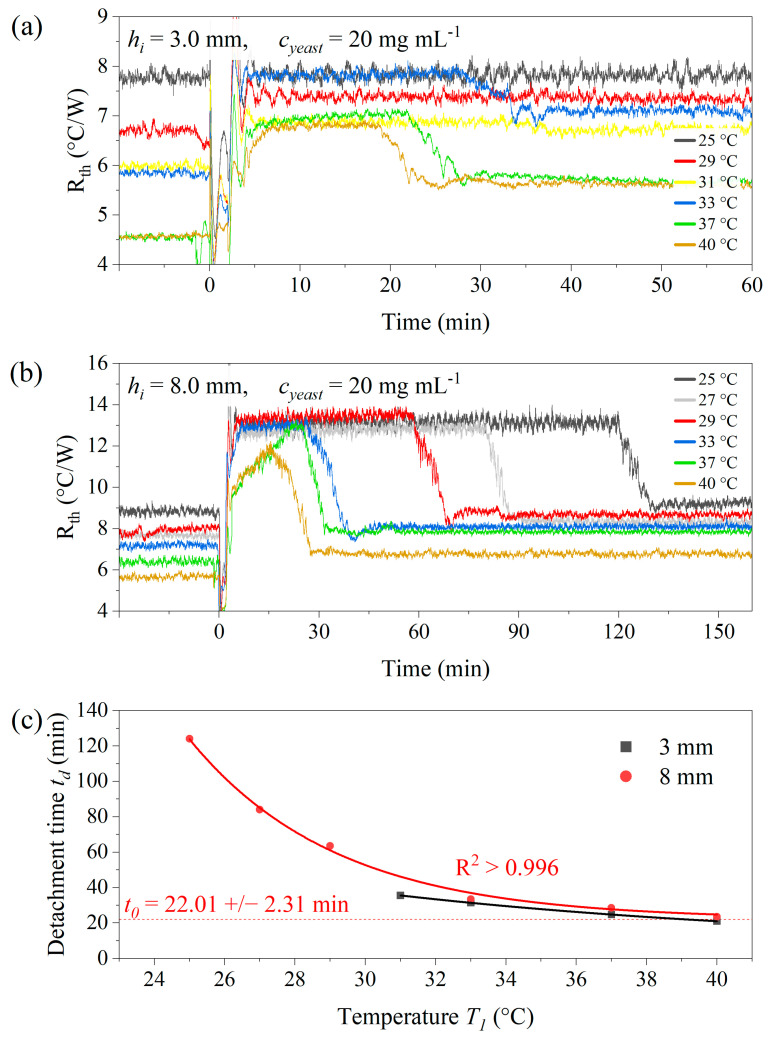
(**a**) Thermal detachment of *S. cerevisiae* for chip temperatures *T*_1_ from 25 up to 40 °C with the inner height of the sample compartment *h_i_* = 3.0 mm and with *h_i_* = 8.0 mm in panel (**b**). The cell concentration in both cases was 20 mg mL^−1^. Panel (**c**) shows *t_d_* as a function of *T*_1_ for both situations, illustrating that the smaller aspect ratio corresponds to slightly reduced dwell times. The solid line through the data for *h_i_* = 8.0 mm is an exponential fit curve based on Equation (6), and *t*_0_ indicates the limit of *t_d_* for high chip temperatures. For *h_i_* = 3.0 mm, no detachment effect could be observed for *T*_1_ < 30 °C. The data points in (**c**) represent individual measurements, and the error bars were determined from a logistic fit based on Equation (7).

In the next set of measurements, a constant concentration of yeast cells was used (*c_yeast_* = 20 mg mL^−1^) in 1 *×* PBS buffer and with the ambient temperature set to *T_a_* = 20.0 °C. Figure 4a shows the *R_th_* data as a function of time for *h_i_* = 3.0 mm, and the corresponding data for *h_i_* = 8.0 mm are included in Figure 4b. The chip temperatures *T*_1_ range from 25 °C up to 40 °C and the start of the sample injection was set as *t* = 0. While detachment can be observed for all *T*_1_ values in the case of *h_i_* = 8.0 mm, there was no identifiable detachment effect for *h_i_* = 3.0 mm when *T*_1_ was below 30 °C. This indicated that convective movement of the fluid media was essential to stimulate the effect. According to computational modeling, when using *h_i_* = 8.0 mm, maximum fluid velocities *v_max_* were between 1.66 mm s^−1^ (*T*_1_ = 25 °C) and 5.02 mm s^−1^ (*T*_1_ = 40 °C). In the case of *h_i_* = 3.0 mm, the *v_max_* values range between 5.41 × 10^−3^ mm s^−1^ and 0.57 mm s^−1^ for the same temperature regime. For an inner height of 3.0 mm, the lowest temperature at which detachment occurred was 31 °C, corresponding to *v_max_* = 0.341 mm s^−1^. This correlates to a maximum shear stress of 9.9 × 10^−4^ Pa. Notably, after detachment, *R_th_* does not fully recover to its baseline value from before the cells were introduced into the compartment. It can be inferred that the presence of the remaining cells continues to contribute to *R_th_*. At lower cell concentrations, *R_th_* after detachment remains indistinguishable from its baseline value [15,18]. Figure 4c shows the detachment times *t_d_* for both inner heights as a function of *T*_1_. While the data for *h_i_* = 3.0 mm span only a narrow temperature range, the empirical fit function Equation (6), adapted from [15,18], accurately represents the data for *h_i_* = 8.0 mm, *R*^2^ > 0.996, as follows:

(6) $$t_{d}\left(T_{1}\right)=t_{0}+A\exp\left(-\frac{T_{1}}{\theta }\right)$$

where *T*_1_ is given in °C, *t*_0_ is the horizontal asymptote, *A* is the amplitude parameter and theta is the scaling factor. The numerical values of the fit parameters are *t*_0_ = 22.0 +/− 2.3 min, *A* = 129.5 +/− 4.0 min and *θ* = 4.2 +/− 0.4 °C. These values were calculated using the Levenberg–Marquardt algorithm, implemented within the Origin software package, 2023b [46]. The absolute values of the parameters are in the same order as in the previous work. While Bakshi Sichani et al. have found a strain dependence of *θ* between different yeast strains, *θ* appears to be influenced by the exact geometry of the sample compartment [18].

Given the objective of this work to develop a technology for drug efficacy testing, the goal is to establish a setting where the detachment time can be derived from the measured data. Therefore, Section 3.5 focuses on an inner height of *h_i_* = 8.0 mm, where detachment was observed at all chip temperatures. Furthermore, the assay time should be short, indicating the need for higher chip temperatures. At *T*_1_ = 37 and 40 °C, detachment had already occurred before the sedimentation was completed, see Figure 4c. In conclusion, *T*_1_ = 30 or 33 °C represents a good compromise between minimizing assay times (*t_d_* < 60 min) and maintaining a highly resolved *R_th_* plateau.

### 3.3. Detachment Effect with Different Cell Concentrations

**Figure 5:**
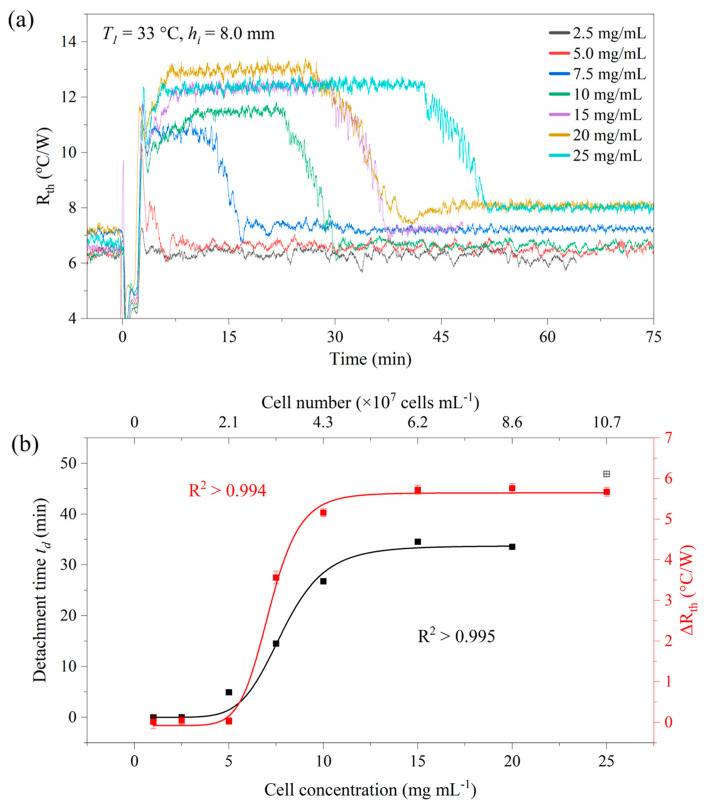
(**a**) Detachment measurements for *h_i_* = 8.0 mm (*Γ* = 0.5) at *T*_1_ = 33 °C and selected cell concentrations from *c_yeast_* = 2.5 mg mL^−1^ to 25 mg mL^−1^. There was no visible detachment for the lowest concentrations, while the height Δ*R_th_* of the plateau is relatively constant for *c_yeast_* > 5.0 mg mL^−1^. (**b**) Dwell time *t_d_* and Δ*R_th_* as a function of the cell concentration (lower *x*-axis) and absolute cell number (upper *x*-axis). Except for 25 mg mL^−1^ in the case of the dwell time *t_d_*, all data scales with a logistic fit according to Equation (6). The data points in (**b**) were determined from panel (**a**) using the logistic fit in the detachment region and uncertainties are smaller than the symbol size.

First, a standard setting was used with *T*_1_ = 33.0 °C and *h_i_* = 8.0 mm (*T_a_* = 20.0 °C). Detachment measurements were performed with cell concentrations *c_yeast_* ranging from 2.5 mg mL^−1^ up to 25 mg mL^−1^. This corresponded to absolute cell numbers between 1.07 × 10^7^ and 1.07 × 10^8^ when taking the volume of the sample compartment into account. The raw *R_th_* data and the corresponding detachment times t_d_ are shown in Figure 5a,b. The trend shows that *t_d_* decreases with lower concentrations and levels off at higher concentrations. However, the situation was different at the lowest concentrations (1.0 and 2.5 mg mL^−1^). Here, no detachment was visible, indicating that detachment happens during the sample injection, resulting in *t_d_* = 0 min. Between 15 and 20 mg mL^−1^, *t_d_* remains effectively constant, making these concentrations advisable for the drug exposure study in Section 3.5. The expected changes in *t_d_* can then be attributed to the effect of the antimicrobial without being influenced by minor uncertainties in the exact cell concentration. For the highest concentration studied, *c_yeast_* = 25 mg mL^−1^, the lengthening of *t_d_* was observed to approximately 50 min, indicating that the assay would take longer than required. Excluding the highest concentration, all data points in Figure 5b follow an empirical logistic fit according to Equation (7):

(7) $$t_{d}=t_{dmax}+\frac{t_{dmin}-{\;t}_{dmax}}{1+{\left(c_{yeast}/c_{yeast}^{’}\right)}^{p}}$$

where *t_d_* is given in minutes, *c_yeast_* and *c*′*_yeast_* in mg mL^−1^, *t_dmin_* and *t_dmax_* the lower and upper horizontal asymptotes, respectively, in minutes and *p* a dimensionless steepness factor with the numerical values of *t_dmin_* ≈ 0 min, *t_dmax_* = 33.7 +/− 1.8 min, *c*′*_yeast_* = 7.8 +/− 0.3 mg mL^−1^ and *p* = 6.9 +/− 1.1. The parameters and their uncertainties were again determined using the Levenberg–Marquard algorithm, and *R*^2^ was 0.995.

### 3.4. Detachment Effect with Different Aspect Ratios

**Figure 6:**
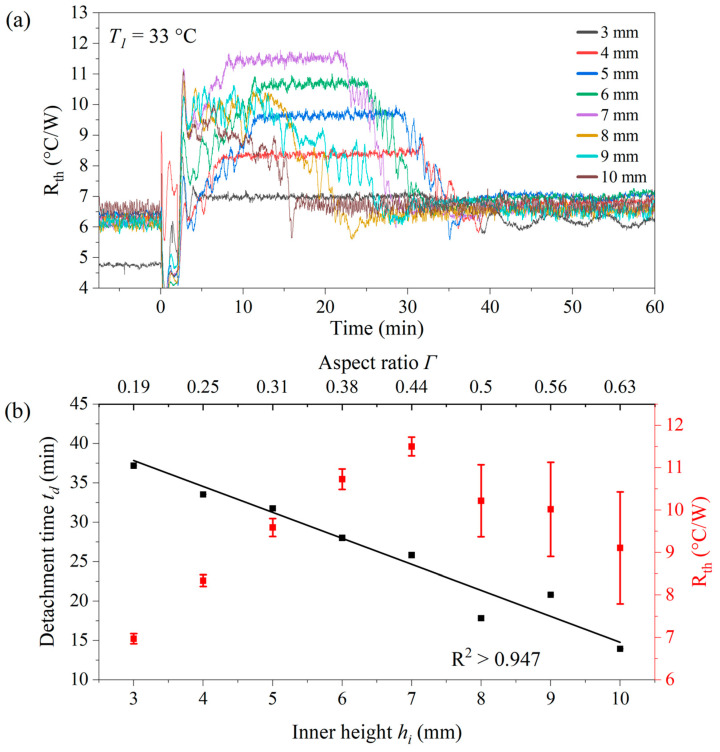
(**a**) Detachment behavior for constant cell numbers *n* = 3.4 × 10^7^ at different inner heights of the sample compartment ranging from 3.0 mm (*Γ* = 0.19) up to 10.0 mm (*Γ* = 0.63) with *T*_1_ = 33.0 °C and *T_a_* = 20.0 °C. (**b**) The detachment time *t_d_* decreases almost linearly for an increasing inner height *h_i_* while the opposite holds for the signal amplitude *R_th_*. Note that for *h_i_* > 7.0 mm, the plateau’s height was no longer sharply defined. Each data point was measured once and the error bar is the uncertainty of the respective measurement; certain error bars are smaller than the symbol size.

In the next series of measurements, the absolute cell number was kept constant at *n* = 3.4 × 10^7^ cells, corresponding to a concentration of 8 mg mL^−1^ when *h_i_* = 8.0 mm. Inner heights ranging from 3.0 to 10.0 mm were selected, with corresponding concentrations decreasing from 21.33 mg mL^−1^ to 6.40 mg mL^−1^ to keep *n* at its predefined value. Figure 6a shows *R_th_* as a function of time for the different inner heights, while the corresponding detachment times *t_d_* and Δ*R_th_* values (height of the plateau over the baseline) are provided in Figure 6b.

Although the decrease in *t_d_* with increasing *h_i_* seems counterintuitive, it is important to note that in all cases, the cells are injected into the sample compartment at a height of 2.0 mm above the chip surface, ensuring that the sedimentation time remains similar. The key difference is in the convective behavior of the fluid, and using COMSOL simulations, maximum fluid velocities ranged from *v_max_* = 0.55 mm s^−1^ for *h_i_* = 3.0 mm up to 3.94 mm s^−1^ in the case of *h_i_* = 8.0 mm at *T*_1_ = 33 °C heating temperatures. Supporting Information S1 provides the corresponding flow patterns and *v_max_* as a function of *h_i_.* This data further supports the idea that convection aids in spontaneous detachment. To reduce assay times, larger inner heights that enhance convection can be used. In Figure 6, at larger inner heights (>7.0 mm), the plateau of *R_th_* was less clearly defined (with a larger uncertainty on Δ*R_th_*). However, the absolute yeast concentrations are low and below the optimum of *c_yeast_* = 15–20 mg mL^−1^ identified in Figure 5 above.

### 3.5. Role of the Temperature Difference T_1_ − T_2_ for the Dwell Time

**Figure 7:**
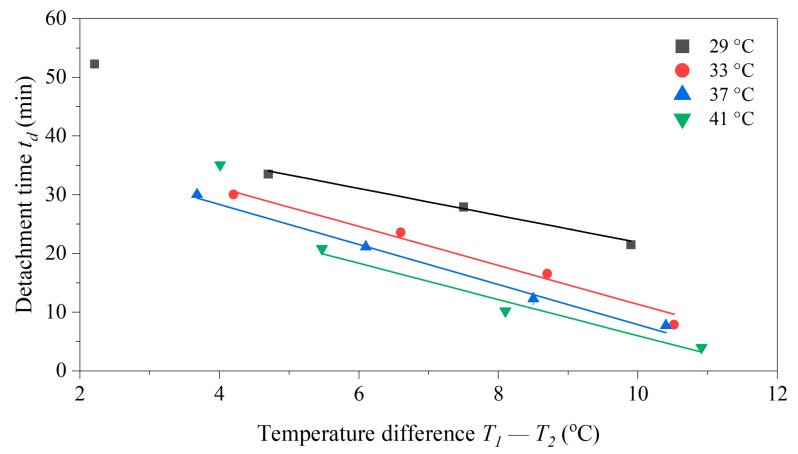
Dwell time *t_d_* for different chip temperatures *T*_1_ = 29, 33, 37 and 41 °C measured at different incubator temperatures *T_a_* of 10, 15, 20, 25 and 30 °C. The combination of *T*_1_ and *T_a_* determines the temperature *T*_2_ inside the sample compartment, and for each data point, the corresponding temperature difference Δ*T* = *T*_1_ − *T*_2_ is indicated. At any given *T*_1_, *t_d_* decreases as Δ*T* increases. Comparing data points with a similar Δ*T* in the linear region shows that *t_d_* decreases with increasing *T*_1_. The inner height was identical for each data point (*h_i_* = 8.0 mm, *Γ* = 0.5) with a cell concentration of 10 mg mL^−1^. Each data point was measured once, and the width of the error bar indicates the uncertainty of each measurement. The error bars are smaller than the symbol size.

To investigate whether thermophoretic forces are involved in the detachment effect, measurements were performed at *T*_1_ = 29, 33, 37 and 41 °C with *h_i_* = 8.0 mm and cell concentration *c_yeast_* = 10.0 mg mL^−1^, see Figure 7. To control the temperature gradient, the ambient temperature provided by the incubator was set to *T_a_* = 15, 20 and 25 °C. The temperature *T*_2_, measured with the thermocouple in the fluid, is then not only determined by *T*_1_ but also by *T_a_*. While the temperature gradient across the yeast layer was difficult to determine, the temperature difference *T*_1_ − *T*_2_ was used to indicate its magnitude. If thermophoresis was the only force at work, then t_d_ would be a unique function of *T*_1_ − *T*_2_ and all data points would align along a single curve. As shown in Figure 7, this was not observed. Although *t_d_* decreases nearly linearly with an increasing temperature difference (also expected in the case of thermophoretic forces), the traces for the different chip temperatures *T*_1_ do not cross. Looking at *T*_1_ − *T*_2_ = 7 °C, *t_d_* was shortest for *T*_1_ = 41 °C and longest for 29 °C. The data does not exclude thermophoretic contributions to the detachment effect, but convection and the biological effects discussed in Section 3.5 must be considered.

### 3.6. Detachment Effect in the Presence of the Antimicrobial Amphotericin B

**Figure 8:**
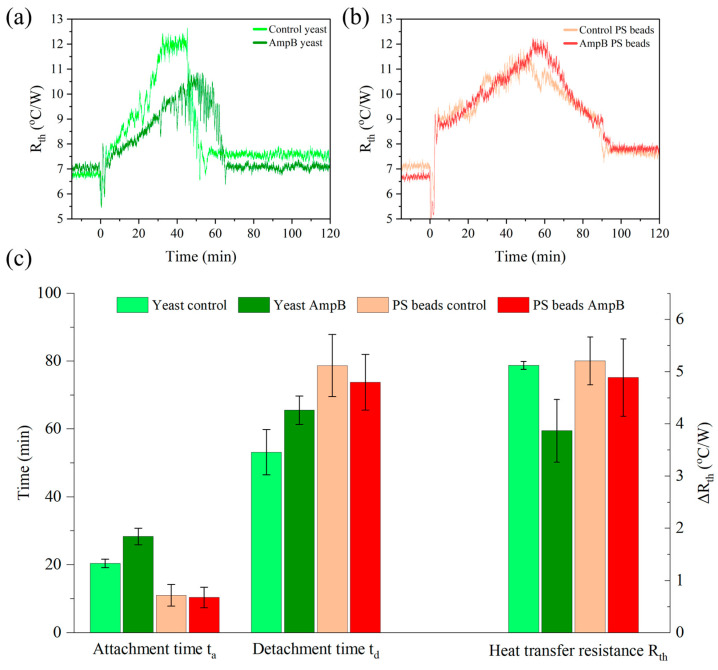
(**a**) Thermal detachment of *S. cerevisiae* (*c_yeast_* = 15 mg mL^−1^, *T*_1_ = 30 °C) in a mixture of 1 × PBS and Milli-Q. Data in dark green refer to cells that were incubated for 48 h with 10 µM AmpB, the negative control (incubated without AmpB) is given in light green. There are apparent differences in the attachment and detachment times, as well as in the magnitude of the *R_th_* change. (**b**) Polystyrene beads incubated at the same conditions show no significant differences between the negative control and the AmpB-treated sample. (**c**) Bar charts of the attachment time *t_a_*, detachment time *t_d_*, and the amplitude of the *R_th_* change for yeast and PS beads with and without exposure to AmpB. All data are averages of three independent replicates with the standard deviation as error bars.

To investigate whether the thermal detachment technique can assess the impact of antimicrobial drugs on microorganisms, yeast suspensions were prepared at a concentration of 15 mg mL^−1^ S. cerevisiae. A mixture of 50 vol.% 1 × PBS and 50 vol.% Milli-Q was used with and without 10 µM AmpB. All samples were incubated for 48 h at 30.0 °C under dark conditions before starting the measurement. The selected yeast concentration corresponds to the region where t_d_ was widely insensitive to *c_yeast_* (see Figure 5b). The measurement parameters were *T*_1_ = 30.0 °C, *T_a_* = 20.0 °C and *h_i_* = 8.0 mm. These settings came with low noise and a clear plateau of the *R_th_* signal, resulting in accurately defined *t_d_* values. Figure 8a presents the corresponding data for *R_th_* as a function of time, clearly showing that *t_d_* for the negative control (53.2 ± 6.7 min) was the shortest, while *t_d_* of the AmpB treated sample has increased to 65.6 ± 4.2 min. A more refined analysis shows that the lengthening of *t_d_* goes along with a lengthening of the attachment time *t_a_* as defined by its 50% value, and the increase of the thermal resistance Δ*R_th_* was less pronounced for the AmpB-treated cells.

Furthermore, to test whether the lengthening of *t_d_* was a biological effect, the experiment was repeated in the same manner, using PS beads instead of yeast cells. The concentration of the PS beads was also chosen as 15 mg mL^−1^ to work under similar conditions. The actual number of particles may differ slightly because of the larger diameter of the PS beads (5.0 µm) compared to yeast cells (3.6 µm). However, this difference was partially offset by the lower mass density of PS (1.05 g cm^−3^) relative to yeast (1.10 g cm^−3^). As seen in Figure 8b, PS also detaches, possibly due to convective shear forces and thermophoresis. However, consistently shorter attachment times *t_a_* and longer detachment times *t_d_* were noted compared to yeast samples. For comparison, Figure 8c shows all data as bar charts (average of three independent replicates). In contrast to yeast, there was no statistically significant difference between the beads exposed to AmpB and those not when comparing the *t_a_*, *t_d_* and Δ*R_th_* data. In conclusion, physical effects must be at work in both cases to make the cells or particles detach. Still, only yeast changes its detachment behavior in response to the antibiotic.

**Figure 9:**
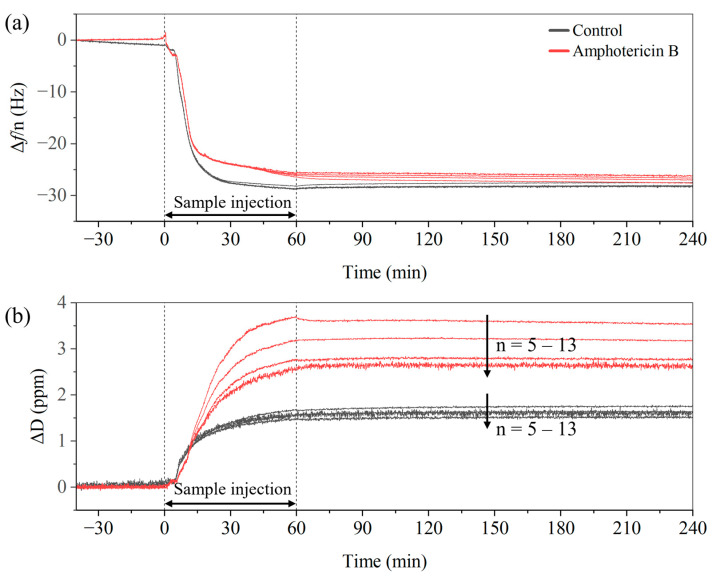
QCM-D measurements on yeast cells (4 mg mL^−1^, 30 °C) in a 1 × PBS-Milli-Q mixture performed after incubation with AmpB (red lines) and without AmpB (black lines). (**a**) The frequency shift Δ*f*/*n* shows no significant differences between the AmpB-treated and untreated cells, the data for different overtones *n* almost coincide. (**b**) The treated cells display a significantly stronger dissipation signal Δ*D* and spreading between the overtones, indicating that the antimicrobial drug enhances the viscoelastic behavior of the cell layer.

To benchmark the HTM-derived results on yeast against an established reference method, two specimens were studied (with and without AmpB) with the dissipation-sensitive quartz–crystal microbalance QCM-D. This technique is well established for analyzing soft-matter interface layers, including cell adhesion and detachment, and has also been used in antimicrobial resistance testing [46,47,48,49]. The samples were prepared the same way as for HTM, see Section 2.5, and the QCM-D was set to 30 °C to resemble the conditions used in the HTM measurement as closely as possible. Figure 9a shows the frequency shift *delta f*/*n* for the overtones *n* = 5 to 13 as a function of time; we could not obtain stable data for the overtones *n* = 1, 3. After the 60 min injection time at a flow rate of 0.1 mL min^−1^, no flow was applied. Samples with and without AmpB show similar values of Δ*f*/*n* with a minimal spreading between the different overtones. Figure 9b shows the time-dependent dissipation signal Δ*D* and we note that Δ*D* of the AmpB-treated yeast was twice as high as for the non-treated version, irrespective of the overtone. In addition, especially for the treated yeast, there was an apparent spreading between the overtones, suggesting that the layer of the treated cells has pronounced viscoelastic properties [50]. This agrees with the observations that AmpB intercalates into the cell walls, making them less rigid. No noticeable detachment effect was observed during the 4 h measurement period using QCM-D. This was expected, as the QCM-D compartments are designed to maintain a uniform temperature distribution without convection effects.

### 3.7. Detachment Effect in the Presence of the Antiseptic Polyvidone Iodine

**Figure 10:**
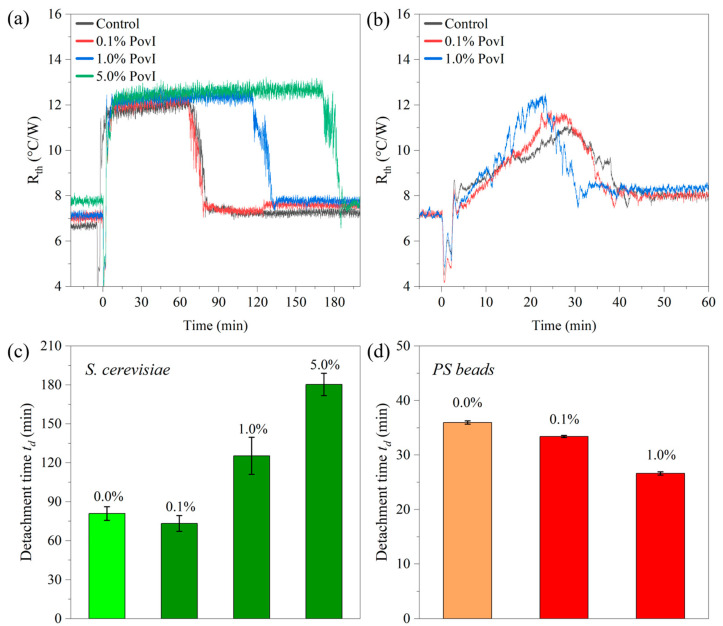
(**a**) Detachment experiment with *S. cerevisiae* (15 mg mL^−1^, *T*_1_ = 30 °C), including a control and 0.1%, 1.0%, and 5% *v*/*v* of PovI. The lengthening of the detachment time was clearly visible at 1% while the drug does not influence the amplitude of the *R_th_* increase. (**b**) Reference measurements on PS beads, exposed to the same PovI concentrations, do not show a lengthening of *t_d_*. (**c**) Bar charts of the detachment time for the different antiseptic drug concentrations for *S. cerevisiae* and (**d**) PS beads. The signals for yeast were calculated from three independent replicates of each experiment, and the error bars represent the standard deviations. Detachment experiments using PS beads with and without PovI were conducted once and the error bars are the uncertainties based on Equation (6).

Finally, the effect of the antiseptic PovI was studied at concentrations of 0.1, 1.0 and 5.0% on the detachment behavior, including a control without the antiseptic agent. As mentioned in Section 2.5, the incubation was only for 30 min at room temperature and the measurement settings were *T*_1_ = 30 °C, *c_yeast_* = 15 mg mL^−1^ and *h_i_* = 8.0 mm. As seen in Figure 10a, the 1.0% dose caused the lengthening of *t_d_* by 45 min while the *R_th_* plateau was well defined. The plateau resembled that of intact yeast cells, see Figure 4 and Figure 5, meaning that iodine does not affect the integrity of cell walls as seen with AmpB. Repeating the measurement with PS microbeads in Figure 10b did not show the lengthening effect, instead detachment seems to accelerate. Tentatively, this can be understood by the density of the PovI solution (measured value 1.04 g mL^−1^), which was already close to the density of the PS beads (1.05 g mL^−1^). At these densities, buoyancy becomes a significant factor. For an easy comparison, Figure 10c,d provides the *t_d_* data of these experiments as bar charts. In conclusion, PovI was more potent in changing the detachment behavior of yeast cells, which was attributed to its fast action mechanisms.

**Figure 11:**
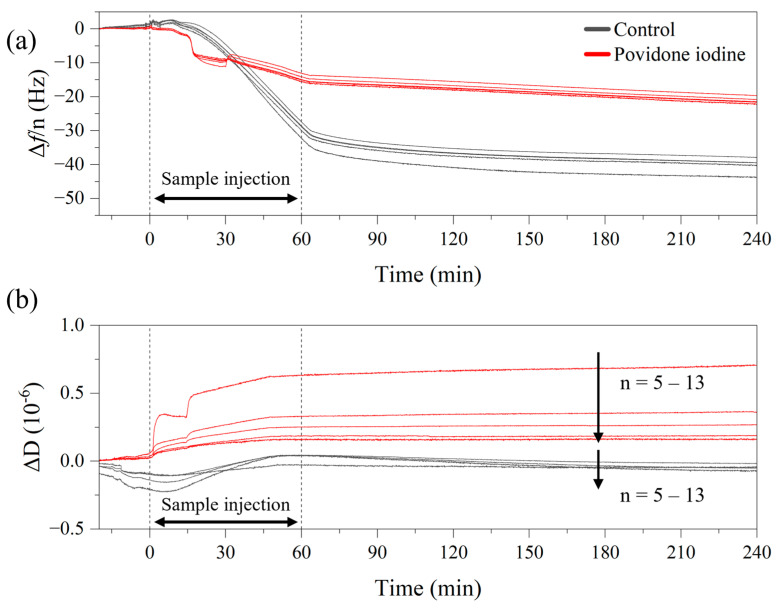
QCM-D measurements on yeast cells (4 mg mL^−1^, 30 °C) in 1 × PBS buffer performed after incubation with 5% PovI (red lines) and without PovI (black lines). (**a**) Exhibits notable differences between PovI-treated and untreated cells, with PovI-treated cells showing reduced mass loading, suggesting lower adhesion to the gold surface. (**b**) The treated cells display a stronger dissipation signal Δ*D* and spreading between the overtones, indicating that PovI promotes a viscoelastic behavior of the cell layer.

To demonstrate that QCM-D was also able to indicate cell damage by PovI, Figure 11a shows the frequency shift Δ*f*/*n* for the control (no PovI) and for 5% *v*/*v* PovI with the QCM-D instrument at *T* = 30 °C and *c_yeast_* = 15 mg mL^−1^. The corresponding dissipation data are provided in Figure 11b.

## 4. Discussion and Conclusions

The spontaneous detachment of eukaryotic cells from the interface between a solid chip and a supernatant aqueous medium under a temperature gradient is governed by several factors. These include the cell type, the cell concentration, the aspect ratio of the sample compartment and the chip temperature. We designed and studied a sample compartment with an adjustable inner height to advance the method toward a valuable tool for in vitro drug testing. In the future, it can potentially serve as an alternative to photonics-based techniques for whole-cell characterization and MIC determination in microorganisms [51,52]. The optimal aspect ratio was approximately 0.5. At lower gamma values, the detachment effect was not observable at chip temperatures below 30 °C, while high gamma values introduced noise on the thermal-resistance signal. The moment of cell detachment, *t_d_*, indicates the cell type under study (in case of an unknown strain). For known strains, changes in *t_d_* can reveal the effects of factors such as nutrients and antimicrobial drugs.

For high-throughput screening, the assay time should be short, ideally less than 60 min, which can be achieved using sufficiently high chip temperatures. Nevertheless, small changes in *t_d_* caused by low drug concentrations must be accurately measurable. Therefore, the uncertainty in determining *t_d_* should be minimal, ideally below 1 min. A compromise between measurement speed and accuracy was achieved with chip temperatures of 29–35 °C. The optimal cell concentration for yeast was between 15 and 20 mg mL^−1^. Higher concentrations resulted in a significant increase in *t_d_*, while at lower concentrations *t_d_* can deviate when a nominal concentration is not met accurately. Furthermore, placing the device inside a temperature-stabilized enclosure enhanced the stability of the thermal-resistance signal. The absolute chip temperature was a contributing factor for detachment, especially considering its role as a partly biologically driven mechanism. With the current device, it was not possible to determine whether convection or thermophoresis is responsible for lifting the cells from the chip surface. Future studies will address this question using more advanced instruments capable of generating shear flow without a temperature gradient or producing a temperature gradient without convective fluid movement.

Regarding future research directions, the sensor device and method can be utilized in several ways and, as it measures the impact of toxic substances on cell cultures, it falls into the category of cell-based biosensors, see Refs. [53,54,55]. We expect that in vitro drug testing will be an important application of the methodology, which is not necessarily limited to yeast cells because spontaneous detachment was already observed on human cell lines [15]. As an initial proof of concept for drug testing, the effects of the antibiotic Amphotericin B and the antiseptic povidone-iodine were studied. The latter led to an extremely retarded detachment even at low doses. Conversely, the method can assess a cell line’s susceptibility to a specific drug. If no susceptibility is noticed, it may serve as an indicator of antimicrobial resistance. From a practical perspective, working with a multiplexed device allowing multiple measurements simultaneously will be promising. Reducing the volume of individual sample compartments could be a viable option for cell types that are difficult to cultivate. Whether miniaturized sample compartments affect the optimal cell concentration, determined in the present work for yeast, needs to be verified. The theoretical limit would be compartments hosting only a single cell and a few research articles are available that address measuring thermal conductivity and heat capacity at the single cell level with advanced methods [56,57]. A current limitation of the approach is the lack of real-time imaging, which limits detailed insight into the detachment dynamics. Future work will address this by integrating live-cell imaging to enhance interpretability and support the reliability of finite element modeling. Additionally, a comprehensive study will compare various cell types and include an in-depth evaluation of drug efficacy for several antibiotics and their concentrations.

## Figures and Tables

**Table 1:** Thermophysical parameters involved in the COMSOL simulations on the temperature and convective velocity profiles inside the sample compartment. *C_p_* is the isobaric heat capacity, *ρ* is the mass density and *κ* is the thermal conductivity. The viscosity of water is *η* = 1.00 × 10^−3^ Pa s. All data refer to 20 °C and were retrieved from Refs. [39,40].

Domain Number	Material	*C_p_* (Jkg^−1^K^−1^)	*ρ* (kgm^−3^)	*k* (Wm^−1^K^−1^)
1	Air	1000	1.12	0.027
2	Copper	385	8700	400
3	PEEK	1700	1320	0.25
4	Stainless steel	445	8900	90.7
5	Teflon	1500	2200	0.25
6	Viton	180	1800	0.104
7	Water	4186	1000	0.62

## Data Availability

The raw data supporting the conclusions of this article will be made available by the authors on request by e-mail to patrickhermann.wagner@kuleuven.be.

## Supplementary Materials

The following supporting information can be downloaded at: https://www.mdpi.com/article/10.3390/s25092902/s1, Figure S1: Parametrization of the sensor device for COMSOL Version 6.2 simulations. Figure S2: Comparison of experimental and computational simulations. Figure S3: Thermal resistance measurements in absence of convection with an inverted device. Figure S4: Optical micrographs of yeast cells with and without exposure to antimicrobials. Figure S5: Metabolic resazurin assay on yeast cells with and without exposure to antimicrobials. Figure S6: HTM measurements using the optimal parameters. Table S1: Maximum velocity of convective flow for different *Γ* values and chip temperatures *T*_1_. Table S2: Contact angles and mass densities of selected sample fluids.
