# Supplementary material for: Spontaneous Cell Detachment from Temperature Gradients: Getting the Method Ready for Antimicrobial Drug Testing at Cell Culture Level
Source: Sensors (Basel). 2025 May 4;25(9):2902. doi: 10.3390/s25092902 (PMC12074233; doi:10.3390/s25092902)
Supplement: Supplementary file 1 [file sensors-25-02902-s001.zip › sensors-3565184-supplementary.pdf]

Supplementary Information

Spontaneous cell detachment from temperature gradients: Getting the method ready for antimicrobial drug testing at cell-culture level

Csongor Tibor Urbán [1], Soroush Bakhshi Sichani [1], Gabriela Ueda Modaffore [1,2], Christ Glorieux [1], Jonas Gruber [2], Derick Yongabi [1], Minne Paul Lettinga [1,3], and Patrick Wagner [1,*]

[1]Laboratory for Soft Matter and Biophysics ZMB, Department of Physics and Astronomy, KU Leuven, Celestijnenlaan 200 D, B-3001 Leuven, Belgium

[2]Departemento de Química Fundamental, Instituto de Química, Universidade de São Paulo, Av. Prof. Lineu Prestes, 748, Butantã - São Paulo, CEP: 05508-000 S.P., Brazil

[3]Biomolecular Systems and Processes IBI-4, Institute of Biological Information Processing, Research Center Jülich, Wilhelm-Johnen-Strasse, D-52428 Jülich, Germany

*Correspondence:

Prof. Dr. Patrick Wagner

KU Leuven, Laboratory for Soft Matter and Biophysics ZMB,
Celestijnenlaan 200 D, B-3001 Leuven, Belgium

Phone: +32 – 16 – 32 21 79
E-mail: PatrickHermann.Wagner@kuleuven.be

Table of contents

SI-1:.........................Finite element simulations

SI-2:.........................Thermal calibration of the HTM device

SI-3:.........................Optical microscopy of yeast cells with and without exposure to antimicrobials

SI-4:.........................Metabolic assay on yeast cells with and without exposure to antimicrobials

SI-5:.........................Contact angles and mass densities of selected sample fluids

SI-6:.........................HTM measurements using the optimal parameters

SI-1 Finite element simulations

To capture realistic thermal behavior, a boundary layer was incorporated at the mid-point of the copper domain (Fig. S1), where the T_1 thermocouple is located. Including the T_2 thermocouple in the simulations did not affect the calculated temperature and velocity distributions. In the computation of the heating temperature ramp for the same geometries, an auxiliary temperature sweep was employed. This allowed each subsequent temperature model to use the previous solution as its starting condition. Within the studied temperature range, the Rayleigh number stays well below the critical threshold to transition into the turbulent convection regime. To simplify the complexity of the Navier-Stokes equations, we used the Boussinesq approximation. Here we assume that density changes are negligible as we deal with small temperature differences and incompressible, low-speed fluids.

For smaller aspect ratios (Γ = 0.1 − 0.4), a stationary study was conducted due to the steady nature of the flow cell. The laminar interface remains suitable within this range. However, at higher aspect ratios, locally turbulent fluid movements can emerge near tight boundaries, even though the overall flow remains predominantly laminar. Consequently, for aspect ratios above this range, a time-dependent study was employed to account for the unsteady nature of the flow in the high-Reynolds number regime. Given that 2D-axisymmetric simulations are computationally efficient, an extremely fine mesh was used relative to the flow cell, consisting of 0.1 mm triangular elements in the fluid domain. We used 1-second steps over the course of 90 minutes.

The temperature of the copper block (domain 2) was set to a predefined temperature T_1 and covered with a stainless-steel chip. A boundary layer was incorporated at the mid-point of the copper domain where the T1 thermocouple is inserted to capture realistic thermal behavior. The flow cell height was parametrically adjusted by varying the aspect ratio, keeping the base radius constant. The modelling of the T_2 thermocouple was excluded to save memory as its inclusion did not significantly affect the isotherms and velocity profiles. Both axes are presented in millimeters. In our simulations, the inner height was parametrically varied from 2 to 8 mm.

Equations 1-5 (see main manuscript) were fully coupled via the non-isothermal laminar flow interface. The laminar flow interface was selected for modelling due to the temperature difference between the bottom and top plates (ambient temperature, T_a = 20 °C) remaining within 13 °C. Within this temperature range, the Rayleigh number stays well below the critical threshold needed to transition into the turbulent convection regime.

The exterior boundary of the flow cell was cooled by applying an outward heat flux through the external natural convection module, which was maintained at an ambient temperature see **Eq. (S1)**. This heat flux was proportional to the temperature difference between the flow cell and its environment, as well as the heat transfer coefficient of the materials used in the flow cell.

$$-\hat{n} = h(T_{ext} - T) \qquad \textbf{Eq. (S1)}$$

where $\hat{n}$ is the surface normal and q is the heat flux (W m^{-2}). The convergence criteria were set with a relative tolerance of 0.001. The nonlinear problem was solved in a fully coupled approach using an iterative PARDISO solver. The simulations were validated using experimental HTM data from baseline measurements with air and Milli-Q water, confirming that the experimental results align with reference values, such as the thermal conductivity of water.

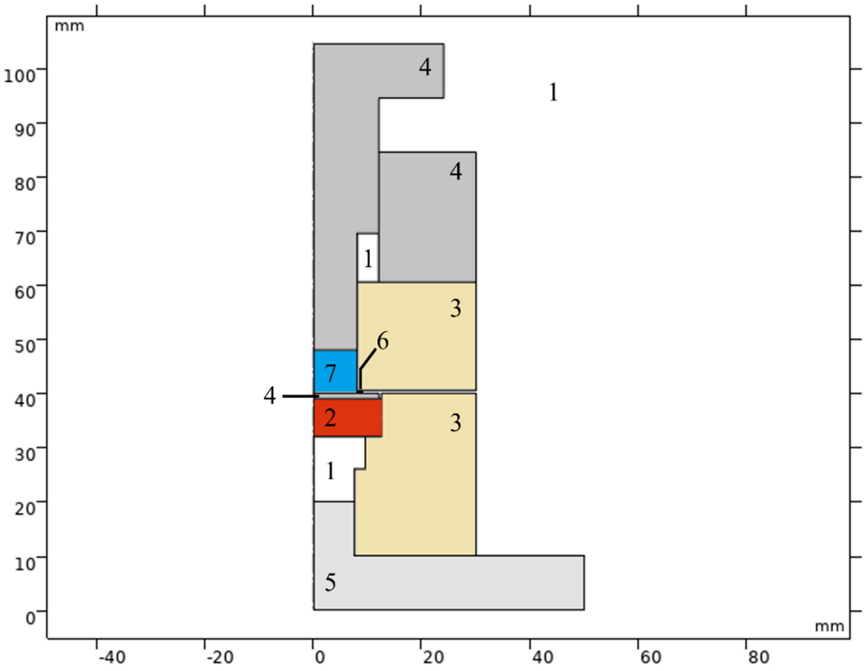

Figure S1: 2D-axisymmetric COMSOL model of the sensor device for finite element simulations.

Table S1: Maximum fluid velocities (mm s^{-1}) of convective flow for different Γ values and chip temperatures T_1. Ambient temperature T_a was always kept at a constant 20 °C.

Inner height h_i (mm)	Aspect ratio Γ	Chip temperature T_1 (°C)			
		21	25	29	33
2	0.125	4.63×10^{-4}	2.62×10^{-3}	5.33×10^{-3}	8.88×10^{-3}
3	0.1875	1.81×10^{-4}	1.01×10^{-3}	0.363	0.549
4	0.25	2.27×10^{-3}	0.673	1.12	1.56
5	0.3125	0.012	0.118	0.155	2.04
6	0.375	0.115	0.14	2.39	3.26
7	0.4375	0.132	1.95	3.28	4.01
8	0.5	0.569	2.27	3.56	3.94

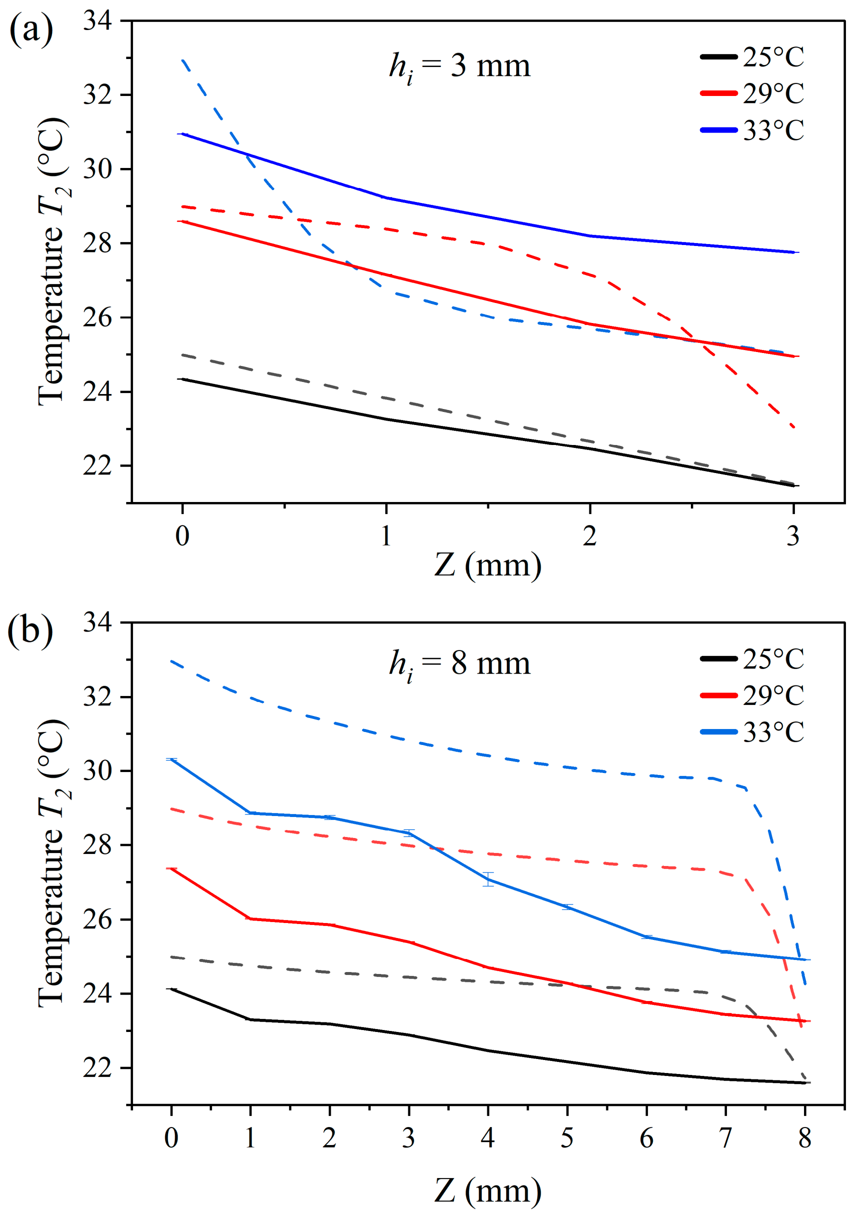

Figure S2: Comparison of experimental and computational simulations. Regular lines represent experimental results, and dashed lines represent computational simulation results. The T_2 thermocouple was used to probe temperatures along the Z-axis. **(a)** Comparison study on 3 mm inner height. **(b)** Comparison study on 8 mm inner height. Increasing both height and temperature can result in deviations from expected flow patterns due to more unpredictable convective fluid movements.

SI-2 Thermal calibration of the HTM device

In this study, R_{th} values were calculated based on the total power applied. However, a portion of the heating power is dissipated in directions that do not cross the chip's solid-fluid interface. The thermal resistance of a cylindrical column can be derived from the following equation:

$$R_{th} = \frac{1}{\kappa}\frac{h}{A} \quad \textbf{Eq. (S2)}$$

where κ is the thermal conductivity (W m^{-1}K^{-1}), h represents the height (m), and A denotes the cross-sectional area of the column (m^2). To minimize convective effects, the experimental setup was inverted. Given a setup height of 10 mm and the T_2 thermocouple positioned 1 mm from the cold plate, thermal resistance was calculated using the literature thermal conductivity value for water (0.624 W m^{-1}K^{-1}). This calculation yields an estimated value of 1.113 W m^{-1}K^{-1} for water, which, while within the same order of magnitude, shows a notable discrepancy attributed to lateral heat dissipation through the walls and base of the setup. The equation $R_{th} = (T_1 - T_2)/P$ does not account for this lateral dissipation, which results in an experimentally determined R_{th} value that underestimates the true thermal resistance. To estimate the power transmitted through the solid-fluid interface accurately, it was assumed that environmental conditions remained consistent between measurements conducted using air and water as the working fluids. A reference measurement was performed using air as the fluid to validate this assumption and adjust for discrepancies. From the data we obtained, we calculated the fraction of air passing through the air column.

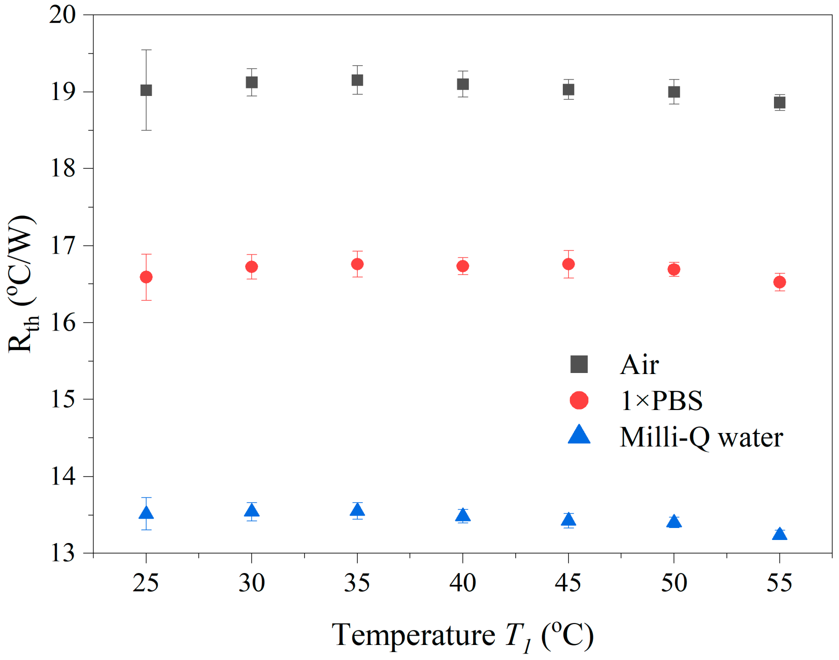

Figure S3: Thermal resistance measurements in the absence of convection with an inverted device.

SI-3 Optical microscopy of yeast cells with and without exposure to antimicrobials

To visualize the yeast cells with and without exposure to AmpB and PovI, images were obtained at room temperature using a widefield microscope (DM 750M, Leica Microsystems, Heerbrugg, Switzerland), coupled with a CCD camera (Leica MC170 HD). For cell imaging, a 100x/0.85 NA objective was used (N Plan Epi 100x/0.85 from the same manufacturer).

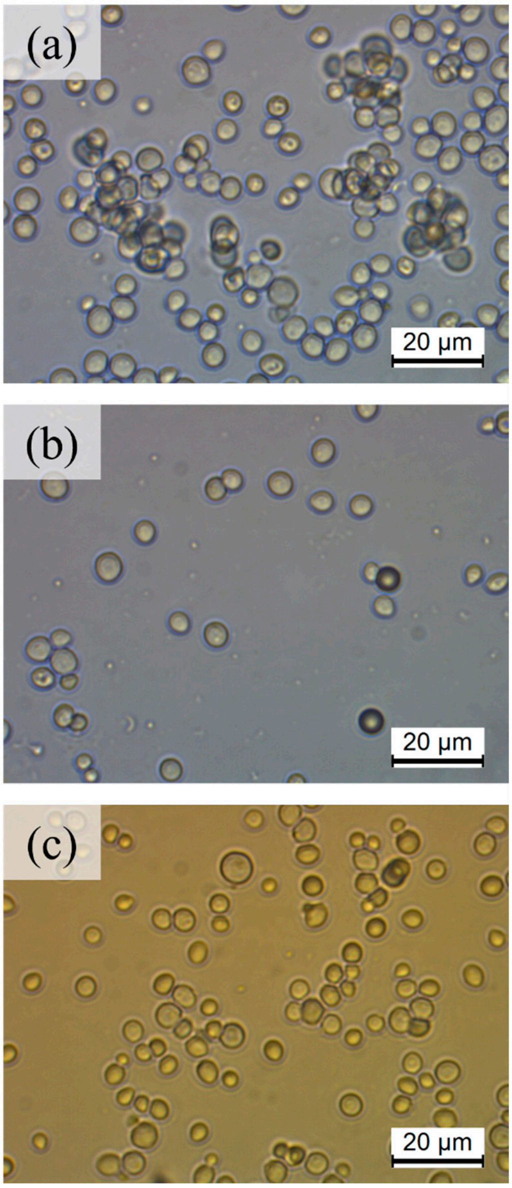

Figure S4: Optical micrographs of yeast cells with and without exposure to microbials. **(a)** Control samples were suspended in 1×PBS buffer. **(b)** Yeast cells treated with 10 μM AmpB. **(c)** Yeast cells treated with 5% v/vPovI. All samples were incubated for 2 hours at 30 °C. Yeast cells exposed to both antimicrobials showed no visible signs of cell damage.

SI-4 Metabolic assay on yeast cells with and without exposure to antimicrobials

A resazurin reagent was used (purity > 99%, Acros Organics, Thermo Fisher Scientific, Geel, Belgium) to assess cell viability. Respiratory reactions in the mitochondria cause the reduction of resazurin (blue color) to resorufin (pink color). The color change serves as an indicator of viable, metabolically active cells. Resazurin solutions (15 μM) were prepared in 1×PBS buffer (pH 7.4). Cell suspensions were incubated at 30 °C for 30 minutes. Treated cells were incubated in 10 μM AmpB or 5% PovI, respectively. After incubation, cells were centrifuged at 2000 × g for 5 minutes at 4 °C. Centrifugation was performed three times and cells were resuspended in 1×PBS buffer. Finally, the cell suspensions were incubated with the resazurin solution (10% v/v) for 6 hours.

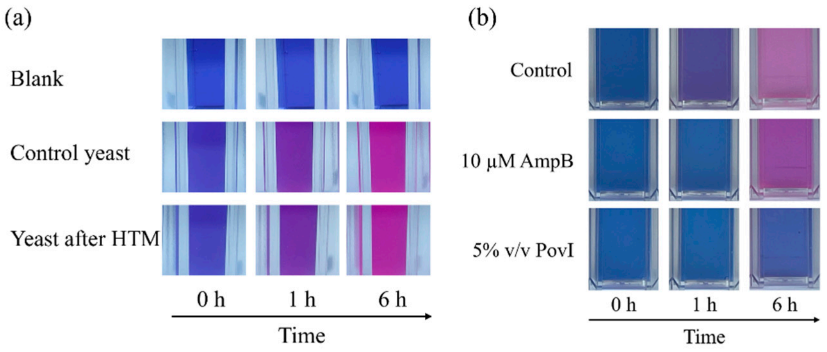

Figure S5: Metabolic resazurin assay on yeast cells with and without exposure to antimicrobials. Untreated control samples showed metabolic activity reflected by the color change after 1 hour of exposure to 10% v/v resazurin solution. **(a)** Viability assay showing yeast cells are metabolically active after HTM measurement at 42 °C. **(b)** After 6 hours of exposure, samples treated with 10 μM AmpB showed decreased viability but remained metabolically active. Samples treated with 5% v/v PovI antimicrobial showed no signs of metabolic activity.

SI-5 Contact angles and mass densities of selected sample fluids

Table S2: Contact angles and mass densities of selected sample fluids. Contact angles were measured using 10 × 10 mm rectangular stainless steel substrates using a contact angle measuring system (DataPhysics, OCA 25, Filderstadt, Germany). Mass densities of the selected fluids were determined under the same conditions using a density meter (Densito, Mettler Toledo N.V., Zaventem, Belgium).

	**Control**	**10 μM AmpB**	**5% v/v PovI**
**Contact angle**	82.1° +/- 9.1°	67.7° +/- 8.4°	66.3° +/- 18.3°
**Density**	1.01 g cm^{-3}	1.01 g cm^{-3}	1.04 g cm^{-3}

SI-6 HTM measurements using the optimal parameters

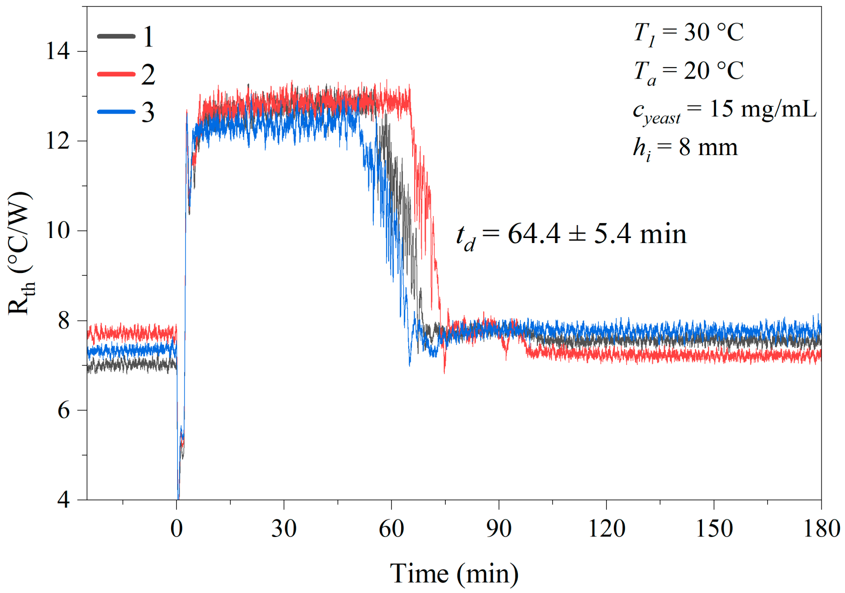

Figure S6: HTM measurements using the optimal parameters.
